# Correction: Predicting art university students’ entrepreneurial intention: A hybrid SEM–ANN approach

**DOI:** 10.1371/journal.pone.0348095

**Published:** 2026-04-27

**Authors:** Yiliang Cao, Jie Zhang

There is an error in affiliation 1 for author Yiliang Cao. The correct affiliation 1 is: College of Liberal Arts, Liaoning University, Shenyang, China.
