# Supplementary material for: Predicting art university students’ entrepreneurial intention: A hybrid SEM–ANN approach
Source: PLoS One. 2025 Aug 21;20(8):e0330833. doi: 10.1371/journal.pone.0330833 (PMC12370118; doi:10.1371/journal.pone.0330833)
Supplement: Attachment — Submitted filename: comments.pdf [file pone.0330833.s002.pdf]

Comment 1

In sections 2.1 and 2.2, use full names ("Diffusion of Innovation Theory" and "Theory of Planned Behavior") in titles instead of acronyms.

Comment 2

The explanation of selected variables in 2.1 should be moved to the methods section. Also, please clarify why certain DOI variables were excluded. If complexity is the issue, specify what kind—statistical, theoretical, or interpretative?

Comment 3

In the TPB section of literature review, specify which universities or types of art schools the students come from. Perhaps discussion of the results from the literature regarding differences in TPB when different students are considered (business school, medicine, law...) Also, consider expanding the TPB discussion from the entrepreneurial intention perspective, as there is rich literature in this domain.

Comment 4

The integration of DOI and TPB is conceptually promising. However, before 2.3, clarify how the two theories are merged given their different dependent variables (intention vs. actual use/adoption). Are these considered synonymous? You decided to make EA (attitudes) depended variable for CO, RA and OB, so this will require some elaboration, as DOI suggests that adoption is depended variable. Also, discuss potential multicollinearity between constructs.

Comment 5

The sentence "Regarding entrepreneurial education, 118 students (43.2%) participated, while 155 students (56.8%) did not" is unclear. Participated in what exactly—formal courses, workshops, or something else?

Comment 6

The list of measurement scales before section 3.3 would be clearer in tabular form: Scale | Source | Example item.

Comment 7

Section 4.1 is overly segmented. Instead of listing validation steps in bullet-point style, rewrite the text as a flowing paragraph with light narrative explanation.

Comment 8

Table 5 is mislabeled—VIF is not a correlation matrix. Please present VIF in standard form: one value per predictor.

Comment 9

The ANN results in section 4.3 are not clearly interpreted. The tables are present, but their meaning and implications remain unclear.

Comment 10

In the discussion, you state "At the same time, RA, CO, and OB are important predictors of attitude.", but your results do not support OB and CO. Later on, you clarify this but this is not correct. Also, clarify how the ranking of predictors (PBC > EA > SN…) was derived—refer to ANN output explicitly.

Comment 11

The discussion should better contextualize results for art students specifically. References used (e.g., Hossain et al., 2021) may not reflect cultural or institutional realities in your sample. A local perspective would strengthen your interpretation.

Comment 12

Theoretical, practical implications and limitations should be integrated into the conclusion section for better flow and structure.

Comment 13

There are several minor English language issues throughout the manuscript. A final proofreading is strongly recommended.
