# Supplementary material for: Predicting art university students’ entrepreneurial intention: A hybrid SEM–ANN approach
Source: PLoS One. 2025 Aug 21;20(8):e0330833. doi: 10.1371/journal.pone.0330833 (PMC12370118; doi:10.1371/journal.pone.0330833)
Supplement: Attachment — Submitted filename: Response to Reviewers.doc [file pone.0330833.s003.doc]

**Editor's comments:**

**Comment 1:**

The title should be shortened - I suggest omitting the second part of the title, since combining TPB and DOI in research of this type is not new

**Response to Comment 1:**

Thank you for your insightful suggestion regarding the title. We fully agree that the combination of TPB and DOI is no longer considered novel in this research domain and that a more concise title would improve readability. In response, we have revised the title to remove the theoretical model references and instead highlight the methodological contribution of our study. The new title reads:

**Predicting Art University Students’ Entrepreneurial Intention: A Hybrid SEM–ANN Approach**

We believe this revised title is shorter, clearer, and more aligned with the methodological focus of the manuscript, while maintaining the core subject matter.

**Comment 2:**

I suggest proofreading the paper to remove spelling and stylistic language errors

**Response to Comment 2:**

Thank you for your comment. We appreciate your suggestion and have thoroughly proofread the manuscript to correct all identified spelling, grammar, and stylistic issues. We have revised sentence structures where necessary to improve clarity, consistency, and academic tone. The entire manuscript has been edited using professional academic English standards to enhance readability and presentation quality.

**Comment 3:**

AMOS is the name of the software, not the data analysis method so that I would exclude it from the list of methods in the introduction

**Response to Comment 3:**

Thank you for pointing this out. We agree that AMOS is a software tool rather than a data analysis method. In response, we have revised the introduction to remove “AMOS” from the list of analytical methods. Instead, we now refer to the method as Structural Equation Modeling (SEM) to reflect the correct terminology. We appreciate your attention to detail, which has helped us improve the accuracy and professionalism of our manuscript.

**Comment 4:**

Figure 1 and Figure 2 should come with the source/reference since the models are not originally yours。

**Response to Comment 4:**

We have added the references for both theoretical models accordingly.

**Comment 5:**

It is necessary to provide insight into the items by which the variables were measured, along with references

**Response to Comment 5:**

Thank you for your valuable suggestion. We have revised the original list of measurement scales into a tabular format to enhance clarity and readability. A summary table has been added before Section 3.3, which includes three columns: scale name, source, and example item. This table has been incorporated into the latest version of the manuscript.

**Comment 6:**

What is shown in Table 5? It is not mentioned in the text at all

**Response to Comment 6:**

The original Table 5 has been renumbered as Table 6 in the revised manuscript. Table 6 presents the results of the VIF test. Moreover, Table 6 has been marked in the manuscript.

**Comment 7:**

The discussion should be more refined, enriched by a better elaboration of the results' similarities and differences with other comparable studies, and with more theoretical implications

**Response to Comment 7:**

Thank you very much for your insightful comment. We fully agree with your suggestion that the discussion should be better contextualized to reflect the specific cultural and institutional background of Chinese art university students. In response, we have substantially revised the relevant sections of the discussion to address this issue.

We enriched our interpretation of Perceived Behavioral Control by referencing Chinese art education practices such as project-based learning, national competitions (e.g., “Internet+ Innovation and Entrepreneurship Competition”), and creative incubation platforms, all of which are common in Chinese art universities and significantly shape students’ entrepreneurial self-efficacy.

For Entrepreneurial Attitude, we added a discussion of government-supported initiatives like the “Double Innovation” policy, and how interdisciplinary entrepreneurship education in Chinese art institutions nurtures a more favorable attitude toward creative entrepreneurship.

Regarding Subjective Norms, we removed the earlier reference to Hossain et al. (2021) and replaced it with a localized explanation grounded in the collectivist cultural context of China, emphasizing the influence of family, mentors, and peer networks in shaping entrepreneurial intentions among Chinese art students.

We also revised our discussion of Entrepreneurial Motivation and Relative Advantage by highlighting the rising importance of cultural and creative industries in China, and how art students’ intrinsic motivations (e.g., autonomy, cultural expression, personal branding) align with these industry trends.

Finally, for the non-significant results related to Compatibility and Observability, we proposed a culturally grounded interpretation: Chinese art students may prioritize self-expression and artistic values over external signals of entrepreneurial compatibility or visibility, which may weaken the effects of CO and OB in this specific population.

**Reviewer 1**

**Comment 1:**

The literature review is thorough but could benefit from more international comparative studies (For instance Pejic Bach, M. et al. (2018). Examining determinants of entrepreneurial intentions in Slovenia: applying the theory of planned behaviour and an innovative cognitive style. Economic research-Ekonomska istraživanja, 31(1), 1453-1471.; Hossain, M. I., Tabash, M. I., Siow, M. L., Ong, T. S., & Anagreh, S. (2023). Entrepreneurial intentions of Gen Z university students and entrepreneurial constraints in Bangladesh. Journal of innovation and entrepreneurship, 12(1), 12.

**Response to Comment 1:**

Thank you very much for your valuable and constructive feedback on our manuscript. We sincerely appreciate your comment regarding the need to include more international comparative studies in the literature review.

In response, we have revised the literature review section to incorporate comparative research findings from different cultural and national contexts. Specifically, we have added the following studies:

(1)Pejic Bach et al. (2018), who examined entrepreneurial intentions among Slovenian students using the Theory of Planned Behavior and innovative cognitive style, highlighting the moderating effects of cultural and cognitive dimensions.

(2) Hossain et al. (2023), who focused on Gen Z university students in Bangladesh and explored entrepreneurial constraints in a developing economy context.

These additions help broaden the theoretical scope and provide a richer comparative understanding of how entrepreneurial intention is shaped across different socio-economic and cultural settings.

**Comment 2:**

explain sample selection criteria

**Response to Comment 2:**

Thank you for your suggestion. We have added a detailed explanation of the sample selection criteria in the methodology section. Specifically, we clarify that participants were full-time art university students who had completed at least one year of study and voluntarily agreed to participate. We also describe the recruitment channels and rationale for choosing this population to ensure relevance to the study's objectives.

**Comment 3:**

The use of convenience sampling from only three universities may limit generalizability. A discussion of sampling limitations should be more prominent in the Discussion section.

**Response to Comment 3:**

Thank you for highlighting this important point. In the revised Discussion section, we have expanded the limitations subsection to more clearly acknowledge the constraints posed by the use of convenience sampling from only three institutions. We now explicitly discuss how this may impact the generalizability of the findings and recommend that future research adopt more diverse and representative sampling strategies.

**Comment 4:**

Common method bias is not address. Adding a procedural or statistical would strengthen the validity.

**Response to Comment 4:**

Thank you very much for your valuable suggestion regarding common method bias. We fully agree that addressing this issue is essential for enhancing the validity of the research findings.

We conducted Harman's single-factor test, and the results showed that the first factor accounted for only 32.4% of the total variance, which is well below the critical threshold of 50%. This indicates that common method bias is not a serious concern in this study.

The above information has been added to the revised manuscript in Section 4.1 Measurement Model.

**Comment 5:**

While SEM-PLS is valid, the rationale for using this method over CB-SEM could be briefly elaborated.

**Response to Comment 5:**

Thank you for your valuable comment regarding the use of SEM-PLS. We agree that clarifying the rationale for choosing this method over CB-SEM is important for methodological transparency.

In response, we have added a detailed explanation in the Data Analysis section of the revised manuscript. Specifically, we chose PLS-SEM over CB-SEM for the following reasons:

(1) PLS-SEM is more appropriate for exploratory research and predictive modeling, which aligns with our study’s objective of identifying key predictors of entrepreneurial intention among art students.

(2) PLS-SEM handles complex models with multiple constructs more flexibly, especially when combining theoretical frameworks (DOI and TPB).

(3) PLS-SEM has fewer assumptions regarding data distribution and sample size. Given our sample of 273 and the lack of multivariate normality, PLS-SEM provides more robust and accurate results in this context.

**Comment 6:**

For those of us not familiar with provinces in China, please state in your paper that Liaoning Province is in China

**Response to Comment 6:**

Thank you for your helpful suggestion regarding geographic clarity. In response, we have revised the manuscript to explicitly state that Liaoning Province is located in China. This clarification has been added to both the Abstract and the Methods section to ensure accessibility for an international audience.

**Comment 7:**

The discussion restates findings well but could be enriched by more theoretical implications, particularly how EI in creative fields differs from technical/business contexts.

**Response to Comment 7:**

Thank you very much for your thoughtful comment regarding the theoretical implications. We agree that distinguishing the nature of entrepreneurial intention in creative versus technical/business contexts is essential for enhancing the academic contribution of this study.

In response, we have revised Section 5.1 Theoretical Implications to elaborate on how EI in creative fields is more identity-driven, intrinsically motivated, and aesthetically oriented, in contrast to the more rational and profit-oriented nature of EI in technical or business domains. This addition aims to expand current theoretical frameworks by emphasizing domain-specific differences in entrepreneurial intention formation.

**Comment 8:**

Consider reflecting on how cultural factors in China may shape EI in ways that might not generalize elsewhere.

**Response to Comment 8:**

Thank you for your helpful suggestion on considering cultural influences. We have added a brief reflection in the Limitations and Future Directions section, noting that cultural factors such as collectivism and Confucian values may shape entrepreneurial intention differently in China compared to other contexts. We also suggest that future studies explore this issue through cross-cultural comparisons.

**Comment 9:**

The language is generally clear, though some minor grammatical issues and awkward phrasing appear.

**Response to Comment 9:**

Thank you for your suggestion. We have carefully proofread the entire manuscript and revised several sentences to improve clarity, grammar, and overall readability. We have also ensured consistency in terminology and corrected minor language issues throughout the text.

**Reviewer 2**

**Comment 1:**

In sections 2.1 and 2.2, use full names ("Diffusion of Innovation Theory" and "Theory of Planned Behavior") in titles instead of acronyms.
**Response to Comment 1:**

Thank you very much for your valuable comments and suggestions, which have helped us further improve the quality and clarity of the manuscript. Based on your feedback, we have made the following revisions:

(1) The original section title “2.1 DOI” has been revised to “2.1 Diffusion of Innovation Theory”;

(2) The original section title “2.2 TPB” has been revised to “2.2 Theory of Planned Behavior”.

These changes enhance the clarity and readability of the section headings and make the manuscript more accessible to a broader academic audience.

**Comment 2:**

The explanation of selected variables in 2.1 should be moved to the methods section. Also, please clarify why certain DOI variables were excluded. If complexity is the issue, specify what kind—statistical, theoretical, or interpretative?
**Response to Comment 2:**

Thank you very much for your valuable comments. In response to your suggestions, we have made the following revisions:

1.We have relocated the explanation regarding the selection of DOI variables (i.e., why we chose “Relative Advantage,” “Compatibility,” and “Observability”) from Section 2.1 to the methodology section. A new subsection titled “3.4 Variable Selection Rationale” has been added to clearly distinguish between theoretical background and research design.

2.Regarding the exclusion of other DOI dimensions such as “Complexity” and “Trialability,” we have provided a detailed explanation in the methodology section. Specifically, the exclusion was based on considerations of both statistical and interpretive complexity:

(1) From a statistical perspective, including all five DOI dimensions would have significantly increased the number of model paths and computational burden, potentially compromising model parsimony and stability—especially given our moderate sample size (n = 273).

(2) From an interpretive standpoint, prior studies (e.g., Venkatesh et al., 2012) have pointed out that in entrepreneurship research—particularly in creative industry contexts—the effects of “Complexity” and “Trialability” are often unstable or unclear. Therefore, we prioritized three core dimensions that have demonstrated stronger empirical validity and more consistent measurement in previous research.

**Comment 3:**	In the TPB section of literature review, specify which universities or types of art schools the students come from. Perhaps discussion of the results from the literature regarding differences in TPB when different students are considered (business school, medicine, law...) Also, consider expanding the TPB discussion from the entrepreneurial intention perspective, as there is rich literature in this domain.
**Response to Comment 3:**

Thank you for your valuable suggestion. In response, we have added a comparative discussion in the TPB section of the literature review to address how TPB constructs may vary across different academic disciplines. Specifically, we noted that business students tend to exhibit higher perceived behavioral control due to structured entrepreneurship education, whereas students in disciplines such as medicine or law often demonstrate lower entrepreneurial attitudes because of fixed or institutionally regulated career pathways.

We also highlighted the distinctive characteristics of art university students. These students are typically driven by intrinsic creativity and personal expression, which may enhance their entrepreneurial attitude but also make them more sensitive to social expectations. Furthermore, in collectivist cultures like China, family and institutional pressures often play a strong role in shaping subjective norms. By incorporating these insights, the study more accurately reflects the contextual and disciplinary dynamics that influence entrepreneurial intention among art university students.

We appreciate this suggestion, which has helped us better situate our study within the broader TPB literature and emphasize its relevance to student populations with diverse academic backgrounds.

**Comment 4:**

The integration of DOI and TPB is conceptually promising. However, before 2.3, clarify how the two theories are merged given their different dependent variables (intention vs. actual use/adoption). Are these considered synonymous? You decided to make EA (attitudes) depended variable for CO, RA and OB, so this will require some elaboration, as DOI suggests that adoption is depended variable. Also, discuss potential multicollinearity between constructs.
**Response to Comment 4:**

Thank you for your insightful and constructive comments. We have addressed your suggestions through the following revisions and clarifications:

1. We added a new paragraph before Section 2.3 to explain the theoretical integration between the Diffusion of Innovation (DOI) theory and the Theory of Planned Behavior (TPB). Although DOI typically focuses on actual adoption behavior, and TPB emphasizes behavioral intention, previous studies have noted that in contexts such as student entrepreneurship—where actual entrepreneurial behavior is difficult to observe—behavioral intention can serve as a valid proxy for adoption behavior (Al Breiki et al., 2023; Modgil et al., 2022). Therefore, in this study, key constructs from DOI (Relative Advantage, Compatibility, and Observability) are treated as antecedents of Entrepreneurial Attitude (EA), which in turn predicts behavioral intention, in line with the TPB framework.
2. Regarding the modeling of DOI constructs as predictors of Entrepreneurial Attitude, we have further clarified the theoretical rationale for this approach: when evaluating entrepreneurial opportunities, university students tend to form positive or negative attitudes based on their subjective perceptions of advantage, compatibility, and visibility. This cognitive evaluation process aligns with the classical decision-making pathway in innovation adoption, and supports the conceptual integration of DOI and TPB.
3. Concerning potential multicollinearity issues, we confirm that this was addressed during the structural model assessment. We tested the Variance Inflation Factor (VIF) for all latent constructs, and all VIF values were below 3, indicating that multicollinearity is not a concern in our model. In addition, we conducted both the Fornell-Larcker criterion and the Heterotrait-Monotrait Ratio (HTMT) to assess discriminant validity. The results met the recommended thresholds, further supporting the independence of the constructs.

**Comment 5:** The sentence "Regarding entrepreneurial education, 118 students (43.2%) participated, while 155 students (56.8%) did not" is unclear. Participated in what exactly—formal courses, workshops, or something else?
**Response to Comment 5:**

Thank you for pointing out this issue. We have revised the sentence to clarify the specific forms of entrepreneurial education in which students participated. Specifically, we now state that students engaged in at least one form of entrepreneurial education, including formal university courses, workshops, lectures, or extracurricular activities related to entrepreneurship. The revised sentence is as follows:

Regarding entrepreneurial education, 118 students (43.2%) reported that they had participated in at least one form of entrepreneurial education, including formal university courses, workshops, lectures, or entrepreneurship-related extracurricular activities; whereas 155 students (56.8%) had not participated in such activities (Table 1).

**Comment 6:** The list of measurement scales before section 3.3 would be clearer in tabular form: Scale | Source | Example item.
**Response to Comment 6:**

Thank you for your valuable suggestion. We have revised the original list of measurement scales into a tabular format to enhance clarity and readability. A summary table has been added before Section 3.3, which includes three columns: scale name, source, and example item. This table has been incorporated into the latest version of the manuscript.

**Comment 7:** Section 4.1 is overly segmented. Instead of listing validation steps in bullet-point style, rewrite the text as a flowing paragraph with light narrative explanation.
**Response to Comment 7:**

Thank you for your suggestion. We have revised Section 4.1 by transforming the original bullet-point format into more coherent narrative paragraphs. Following the recommendations of Hair (2021), the revised version presents the validation steps of the measurement model using fluent language and appropriate explanatory content. This modification has improved the readability and academic professionalism of the results section.

**Comment 8:** Table 5 is mislabeled—VIF is not a correlation matrix. Please present VIF in standard form: one value per predictor.

**Response to Comment 8:**

Thank you very much for your comment. We fully understand your concern regarding the format of Table 5. Here, we would like to clarify that our original presentation follows the recommendation of Hair (2021), in which VIF values in PLS-SEM models are displayed in a matrix format, with each endogenous variable corresponding to the VIF values of its predictor constructs. This format is commonly used in SmartPLS outputs and is particularly suited for examining multicollinearity in complex models.

Nonetheless, we fully acknowledge your suggestion to adopt a more traditional and simplified presentation. Therefore, we have revised Table 6 using the standard format in which each predictor construct is reported with a single VIF value—an approach more consistent with conventional regression analysis and CB-SEM literature. This revision improves the clarity of the table and aligns more closely with the presentation norms of mainstream academic journals.

**Comment 9:**
The ANN results in section 4.3 are not clearly interpreted. The tables are present, but their meaning and implications remain unclear.

**Response to Comment 9:**	Thank you for your valuable comment. We fully agree that clear interpretation is essential for understanding the ANN results. In the revised manuscript, we have added a narrative explanation at the end of Section 4.3 to clarify the meaning of the normalized importance values derived from the sensitivity analysis. Specifically, we have highlighted the relative influence of each predictor and explained that Perceived Behavioral Control (PBC) and Entrepreneurial Attitude (EA) are the most important variables in predicting entrepreneurial intention within the ANN model. We also added a cross-reference to Section 5, where the implications and theoretical significance of these findings are further elaborated. This revision improves the interpretability and relevance of the ANN results.

**Comment 10:** In the discussion, you state "At the same time, RA, CO, and OB are important predictors of attitude.", but your results do not support OB and CO. Later on, you clarify this but this is not correct. Also, clarify how the ranking of predictors (PBC > EA > SN...) was derived—refer to ANN output explicitly.

**Response to Comment 10:**	Thank you for raising this important point. We would like to clarify that the statement “RA, CO, and OB are important predictors of attitude” was originally intended to refer to the research hypotheses proposed based on prior literature, rather than the empirical findings of this study. We recognize that presenting this sentence in the discussion section may have led to confusion for readers. Therefore, we have revised the wording to explicitly indicate that these relationships are part of the theoretical hypotheses, not supported results from our data analysis. This revision helps to clearly distinguish between the proposed hypotheses and the empirical evidence.

**Comment 11:**	The discussion should better contextualize results for art students specifically. References used (e.g., Hossain et al., 2021) may not reflect cultural or institutional realities in your sample. A local perspective would strengthen your interpretation.
**Response to Comment 11:**

Thank you very much for your insightful comment. We fully agree with your suggestion that the discussion should be better contextualized to reflect the specific cultural and institutional background of Chinese art university students. In response, we have substantially revised the relevant sections of the discussion to address this issue.

We enriched our interpretation of Perceived Behavioral Control by referencing Chinese art education practices such as project-based learning, national competitions (e.g., “Internet+ Innovation and Entrepreneurship Competition”), and creative incubation platforms, all of which are common in Chinese art universities and significantly shape students’ entrepreneurial self-efficacy.

For Entrepreneurial Attitude, we added a discussion of government-supported initiatives like the “Double Innovation” policy, and how interdisciplinary entrepreneurship education in Chinese art institutions nurtures a more favorable attitude toward creative entrepreneurship.

Regarding Subjective Norms, we removed the earlier reference to Hossain et al. (2021) and replaced it with a localized explanation grounded in the collectivist cultural context of China, emphasizing the influence of family, mentors, and peer networks in shaping entrepreneurial intentions among Chinese art students.

We also revised our discussion of Entrepreneurial Motivation and Relative Advantage by highlighting the rising importance of cultural and creative industries in China, and how art students’ intrinsic motivations (e.g., autonomy, cultural expression, personal branding) align with these industry trends.

Finally, for the non-significant results related to Compatibility and Observability, we proposed a culturally grounded interpretation: Chinese art students may prioritize self-expression and artistic values over external signals of entrepreneurial compatibility or visibility, which may weaken the effects of CO and OB in this specific population.

These revisions provide a more nuanced and culturally relevant interpretation of our findings and significantly improve the alignment between the discussion and our sample context. We appreciate your recommendation and believe this change has enhanced the clarity, applicability, and academic value of the manuscript.

**Comment 12:**
Theoretical, practical implications and limitations should be integrated into the conclusion section for better flow and structure.
**Response to Comment 12:**

Thank you for this thoughtful suggestion. We fully understand and appreciate the rationale behind integrating the theoretical implications, practical contributions, and study limitations into the conclusion to improve flow and readability.

However, after careful consideration, we have decided to retain a more conventional structure in which each of these components is presented in a distinct subsection. This approach follows the organizational style commonly used in prior studies published in leading journals (e.g., Journal of Business Venturing, Entrepreneurship Theory and Practice, Technological Forecasting and Social Change) (Canestrino et al., 2025; Miroshnychenko et al., 2021), where separating these elements allows for greater clarity and depth of discussion.

We believe that this structure enables readers to more easily locate and interpret the theoretical contributions, practical insights, and limitations/future directions independently, without conflating their purposes. Nevertheless, we have ensured smooth transitions between these subsections to maintain coherence and narrative flow.

We respectfully hope this rationale is acceptable and thank you again for your valuable input.

**Comment 13:**
There are several minor English language issues throughout the manuscript. A final proofreading is strongly recommended.

**Response to Comment 13:**

Thank you for your suggestion. We have carefully proofread the entire manuscript and revised several sentences to improve clarity, grammar, and overall readability. We have also ensured consistency in terminology and corrected minor language issues throughout the text.
