# Supplementary material for: Predicting art university students’ entrepreneurial intention: A hybrid SEM–ANN approach
Source: PLoS One. 2025 Aug 21;20(8):e0330833. doi: 10.1371/journal.pone.0330833 (PMC12370118; doi:10.1371/journal.pone.0330833)
Supplement: Attachment — Submitted filename: Response to Reviewer.docx [file pone.0330833.s004.docx]

**Response to Reviewer’s Comment**

**Reviewer Comment 1:** “…factor ’ s relative importance. - part of the abstract, unnecessary space before s.”

**Response:** Thank you for pointing out this typographical issue in the abstract. We have carefully corrected the spacing error by removing the unnecessary space before the apostrophe **’s** in “…factor’s relative importance.” In addition, we conducted a meticulous line-by-line proofreading of the entire manuscript to identify and correct any other spacing or punctuation inconsistencies. This ensures alignment with the PLOS ONE criteria regarding clear, correct, and unambiguous English.

Furthermore, as PLOS ONE emphasizes the importance of professional language presentation, we have revised the manuscript thoroughly for standard academic English style and clarity. Where necessary, we also consulted professional editorial support to enhance the overall language quality.

We sincerely appreciate this helpful observation.

**Reviewer Comment 2:** *“Between the text and the parentheses in which the reference or abbreviation is listed, a space is required.”*

**Response:** Thank you for bringing this formatting issue to our attention. In accordance with your suggestion and the formatting standards required by PLOS ONE, we have carefully reviewed the entire manuscript and inserted a space between the preceding text and all parentheses containing references or abbreviations (e.g., “…as suggested by previous studies (Smith et al., 2022)” → “…as suggested by previous studies (Smith et al., 2022)”).

We have ensured that this spacing adjustment has been applied consistently throughout the manuscript to maintain professional presentation and alignment with the journal’s formatting expectations.

**Reviewer Comment 3:** *“The structure of this paper is as follows: The second section presents the literature review and research hypotheses. After the word follows a dot is needed, not a colon.”*

**Response:** Thank you for identifying this punctuation inconsistency. In accordance with your suggestion, we have revised the sentence by replacing the colon with a period after the phrase “The structure of this paper is as follows.” The corrected sentence now reads:

“The structure of this paper is as follows. The second section presents the literature review and research hypotheses…”

We have also carefully reviewed the manuscript for similar punctuation issues and have ensured consistent and proper use of colons and periods throughout. Your attention to detail is greatly appreciated and has helped us improve the clarity and formal presentation of the paper.

**Reviewer Comment 4:***“The TPB has five key constructs: attitude, SN, perceived behavioral control (PBC), behavioral intention, and actual use (Fig. 2). Why is SN in the abbreviation, and the rest is not? The style should be consistent.”*

**Response:** Thank you for your thoughtful comment regarding the consistency of abbreviations in our description of the Theory of Planned Behavior (TPB) constructs. We would like to clarify that, in this instance, we intentionally did not abbreviate “attitude” and “behavioral intention” because these constructs are not directly applied in our study and are introduced only as part of the theoretical background. In contrast, both “subjective norm (SN)” and “perceived behavioral control (PBC)” are used not only within the TPB framework but also directly adopted in our research model, where their abbreviated forms are consistently applied.

Moreover, “SN” was previously introduced and abbreviated in an earlier section, which is why it appears in abbreviated form here. We have ensured that the usage remains consistent throughout the manuscript based on whether the construct is contextually relevant to our model or mentioned only in the theoretical overview.

We appreciate your attention to this detail and hope this explanation clarifies our rationale for the selective use of abbreviations.
